# Myeloperoxidase Enhances Etoposide and Mitoxantrone-Mediated DNA Damage: A Target for Myeloprotection in Cancer Chemotherapy[Fn FN3]

**DOI:** 10.1124/mol.116.106054

**Published:** 2017-01

**Authors:** Mandeep Atwal, Emma L. Lishman, Caroline A. Austin, Ian G. Cowell

**Affiliations:** Institute for Cell and Molecular Biosciences, Newcastle University, Newcastle upon Tyne. United Kingdom

## Abstract

Myeloperoxidase is expressed exclusively in granulocytes and immature myeloid cells and transforms the topoisomerase II (TOP2) poisons etoposide and mitoxantrone to chemical forms that have altered DNA damaging properties. TOP2 poisons are valuable and widely used anticancer drugs, but they are associated with the occurrence of secondary acute myeloid leukemias. These factors have led to the hypothesis that myeloperoxidase inhibition could protect hematopoietic cells from TOP2 poison-mediated genotoxic damage and, therefore, reduce the rate of therapy-related leukemia. We show here that myeloperoxidase activity leads to elevated accumulation of etoposide- and mitoxantrone-induced TOP2A and TOP2B-DNA covalent complexes in cells, which are converted to DNA double-strand breaks. For both drugs, the effect of myeloperoxidase activity was greater for TOP2B than for TOP2A. This is a significant finding because TOP2B has been linked to genetic damage associated with leukemic transformation, including etoposide-induced chromosomal breaks at the *MLL* and *RUNX1* loci. Glutathione depletion, mimicking in vivo conditions experienced during chemotherapy treatment, elicited further MPO-dependent increase in TOP2A and especially TOP2B-DNA complexes and DNA double-strand break formation. Together these results support targeting myeloperoxidase activity to reduce genetic damage leading to therapy-related leukemia, a possibility that is enhanced by the recent development of novel specific myeloperoxidase inhibitors for use in inflammatory diseases involving neutrophil infiltration.

## Introduction

Drugs targeting DNA topoisomerase II (TOP2 poisons) are important, effective, and widely used anticancer agents, but they are associated with short- and long-term toxic side effects, including neutropenia and rare but life-threatening therapy-related acute myeloid leukemia (t-AML) (Allan and Travis, 2005; Leone et al., 2010; Cowell and Austin, 2012). As cancer survival rates have increased, t-AML has become a more important clinical problem, and it is estimated that up to 15% of all acute myeloid leukemia cases can be classified as t-AML (Mauritzson et al., 2002). Therapy-related acute leukemias occur after a wide range of primary neoplasias, but prior treatment of breast cancer accounts for about 50% of cases, while hematological malignancies account for approximately 30% (Kayser et al., 2011). In its normal cellular role, TOP2 facilitates changes to DNA topology by allowing one double stranded segment to pass through another via an enzyme-bridged DNA double-strand break (DSB) (Austin and Marsh, 1998; Vos et al., 2011; Cowell and Austin, 2012). In this configuration, each protomer of the homodimeric TOP2 enzyme is covalently coupled to a cleaved DNA strand via a 5′-phosphotyrosine linkage. TOP2 poisons such as etoposide and mitoxantrone exert their tumoricidal effect by stabilizing this normally transient enzyme-bridged break, resulting in the accumulation of cytotoxic covalently linked TOP2 protein-DNA complexes, which can be processed in the cell to DNA double-strand breaks (Burma et al., 2001; Cowell and Austin, 2012; Lee et al., 2012, 2016). Therapy related leukemias, especially those appearing after exposure to TOP2 poisons often contain recurrent chromosome translocations, including *t(15,17)(PML-RARA)*, *t(8,21)(AML-ETO)*, and *11q23* rearrangements involving the *MLL* gene (Rowley and Olney, 2002; Cowell and Austin, 2012). These genetic lesions disrupt blood cell development and play a pivotal role in the development of the disease. Such t-AML cases arise as a result of TOP2 poison-mediated DNA damage in bone marrow blood precursor cells. There are two vertebrate TOP2 paralogues, TOP2A and TOP2B; TOP2 poisons such as etoposide affect both paralogues, but recent evidence points to a greater role for TOP2B in generating the genotoxic damage associated with TOP2 poisons (Azarova et al., 2007; Cowell et al., 2012; Smith et al., 2014a).

We are interested in why cells of the myeloid hematopoietic lineage are sensitive to TOP2 poison-mediated genotoxic damage, which leads to t-AML, and how this sensitivity could be reduced. Myeloperoxidase is expressed exclusively in cells of the myeloid lineage; it is present at high levels in neutrophils where it exerts its antimicrobial action but is also expressed in myeloid precursor/progenitor cells, including human and mouse common myeloid progenitor and granulocyte/macrophage progenitor cells (Strobl et al., 1993; Mori et al., 2009; Goardon et al., 2011) and is readily detectable in ex vivo normal human bone marrow CD34^+^ cells (Supplemental Fig. 1) (Strobl et al., 1993; Vlasova et al., 2011). Thus, MPO is likely to be present in the cells in which t-AML arises. In its physiologic role MPO generates hypochlorous acid from hydrogen peroxide and chloride ions to kill pathogenic microorganisms. However, MPO activity also leads to the oxidative activation of etoposide. This occurs by one-electron oxidation of the etoposide E-ring, yielding a phenoxy radical species and by *O*-demethylation to the reactive orthoquinone (Supplemental Fig. 2A) (Haim et al., 1986; Kalyanaraman et al., 1989; Kagan et al., 2001; Fan et al., 2006; Jacob et al., 2011; Vlasova et al., 2011). Additionally, CYP3A4 and/or CYP3A5 can oxidize etoposide to etoposide catechol, a metabolite found in patient plasma (Zheng et al., 2004; Zhuo et al., 2004), and oxidation of etoposide by CYP3A4 has been implicated in the incidence of t-AMLs (Felix et al., 1998). Etoposide catechol can be oxidized to etoposide quinone by MPO via a semiquinone species (Supplemental Fig. 2A). Etoposide quinone is more effective at inducing TOP2-mediated DNA breaks than etoposide in vitro (Jacob et al., 2011) and this is particularly true for TOP2B (Smith et al., 2014b). This increased potency of etoposide quinone as a TOP2 poison has been attributed to covalent modification of the enzyme by the quinone (Jacob et al., 2011; Smith et al., 2014b). Furthermore, etoposide metabolites have the potential to form adducts with DNA and protein (Haim et al., 1987; Lovett et al., 2001), which may also impact on genotoxicity.

Oxidative activation of TOP2 poisons is not limited to etoposide because MPO has been implicated in the activation of mitoxantrone either directly or via the production of reactive aldehydes (Panousis et al., 1995, 1997; Hazen et al., 1998; Parker et al., 1999; Evison et al., 2016).

Using pharmacological inhibition and genetic manipulation we show that MPO activity increases etoposide and mitoxantrone-induced formation of TOP2A and TOP2B covalent enzyme-DNA complexes in cells of myeloid origin and similarly increases DSB formation. GSH depletion amplified this effect. This is consistent with the hypothesis that MPO inhibition, in a clinical setting, especially where cellular thiol levels are suppressed, could protect against genotoxic damage in the myeloid compartment by TOP2 poisons.

## Materials and Methods

### 

#### Reagents and Antibodies.

Etoposide, mitoxantrone, succinylacetone (SA), dimethyl sulfoxide, Tween 20, Triton X-100, paraformaldehyde, dl-buthionine sulfoximine and PF-1355 (2-(6-(2,5-dimethoxyphenyl)-4-oxo-2-thioxo-3,4-dihydropyrimidin-1(2H)-yl)acetamide) were purchased from Sigma-Aldrich (Dorset, UK). MPOi-II (4-(5-fluoro-1H-indol-3-yl)butanamide) was purchased from Merck-Millipore (Watford, UK). Anti-MPO ab9535 (immunofluorescence) and ab134132 (Western blotting) were from Abcam (Cambridge UK), anti-mouse *γ*H2AX 05-636 was obtained from Merck-Millipore.

#### Cell Culture.

All cell lines were maintained in RPMI-1640 medium supplemented with 10% fetal bovine serum and 1% penicillin and streptomycin (Thermo Fisher Scientific, Paisley, UK). Cells were cultured at 37°C in a humidified atmosphere containing 5% CO_2_. Experiments were conducted on cells growing in log phase (2–5 × 10^5^cells/ml). For succinylacetone treatment of cells to downregulate MPO, cells were treated with 200 *μ*M SA for 48 hours before addition of TOP2 poison and downstream analysis. SA was retained during TOP2 poison treatment.

#### Stable Transfection of K562 Cells with MPO cDNA.

K562 cells were transfected with plasmid RC2016029 containing the MPO coding sequence with a C-terminal MYC-DDK tag in the vector pCMV 6-Entry (Origene, Rockville, MD). Transfection was performed using Dharmafect Duo (Dharmacon, Amersham, UK) transfection reagent. Transfection mixtures contained 1 *µ*g/ml of MPO plasmid (Origene) or G418 control plasmid with 20 *µ*g/ml of Dharmafect Duo (Dharmacon) and 80% v/v serum-free RPMI 1640 medium. After selection with G418 (750 *μ*g/ml), clonal lines were isolated by limited dilution. MPO-expressing lines were continuously grown in 500 *µ*g/ml G418, the selection antibiotic. MPO expression was assessed using immunofluorescence and MPO activity assays.

#### MPO and GSH Colorimetric Activity Assays.

MPO activity assays were performed using an Abcam MPO Activity Assay kit (ab105136, Abcam). Cells were harvested by centrifugation at 1000 *g* for 5 minutes and homogenized in 4 pellet volumes of lysis buffer provided. Bradford assays were conducted to ensure an equal concentration of protein was used for each assay. The MPO activity assay was conducted according to manufacturer’s instructions with absorbance readings measured at 415 nm. GSH assays were performed using a GSH assay kit (KA0797, Abnova, Taipei City, Taiwan), according to the manufacturer’s instructions.

#### Immunoblotting for MPO.

Whole cell lysates of cells were prepared (Mirski et al., 1993) and samples were resolved on precast 4–20% SDS-polyacrylamide gels (NuSep, Wasserburg, Germany). Western blotting was performed by standard methods using ECL detection.

#### Trapped in Agarose DNA Immunostaining Assay.

Trapped in agarose DNA immunostaining (TARDIS) assays to quantify TOP2 covalent protein-DNA complexes were carried out essentially as described previously (Willmore et al., 1998; Cowell et al., 2011b). Briefly cells were treated for 1 hour with the desired dose of etoposide or mitoxantrone. Cells were then pelleted and washed in ice-cold PBS. After being recentrifuged, cells were mixed with molten 2% LMP agarose (Lonza, Basel, Switzerland) in PBS at 37°C and spread evenly onto agarose coated slides. Agarose-embedded cells were lysed in (1% w/v SDS, 20 mM sodium phosphate, 10 mM EDTA, pH 6.5) and noncovalently DNA bound proteins were removed using 1 M NaCl. TOP2 covalent complexes were detected by immunofluorescence using rabbit anti-TOP2A (4566) or anti-TOP2B (4555) antibodies, raised to the C-terminal domain of human TOP2A and TOP2B, respectively, and Alexa-488 coupled anti-rabbit secondary antibodies (Thermo Fisher Scientific). Slides were counterstained with Hoechst 33258 to visualize DNA. Quantitative immunofluorescence was performed by capturing images for Hoechst and Alexa-488 using a epifluorescence microscope (Olympus IX-81, Olympus, Tokyo, Japan) fitted with an Orca-AG camera (Hamamatsu, Tokyo, Japan) and suitable narrow band filter sets. Images were analyzed using Volocity 6.3 (Perkin Elmer, Waltham, MA), and data representation and statistics were performed using GraphPad Prism 4.0 and R (San Diego, CA).

#### Immunofluorescence Analysis of *γ*H2AX and MPO.

After drug treatment cells were washed and pelleted in ice-cold PBS and spotted onto poly-l-lysine slides. Cells were fixed in 4% formaldehyde in PBS and permeabilized using KCM+T buffer (120 mM KCl, 20 mM NaCl, 10 mM Tris-HCl pH 8.0, 1 mM EDTA, 0.1% Triton X-100). After blocking in (KCM+T, 2% bovine serum albumin, 10% dry milk powder) cells were probed with primary anti-MPO antibody or anti-*γ*H2AX in blocking buffer and Alexa-488 anti-rabbit or Alexa-594 anti-mouse secondary antibodies (Thermo Fisher Scientific). Slides were counterstained with DAPI (4’,6-diamidino-2-phenylindole) (Vector Laboratories, Burlingame, CA) and viewed using an epifluorescence microscope (Olympus IX-81). For *γ*H2AX quantification, images were captured for DAPI and Alexa-594 and quantitative analysis was performed using Volocity 6.3 (Perkin Elmer) with data representation performed in GraphPad Prism 4.0 and R.

#### GSH Depletion.

NB4 cells were treated with 150 *μ*M buthionine sulfoximine (BSO) for 4.5 hours (Griffith, 1982; Gantchev and Hunting, 1997) before addition of TOP2 poison. BSO was retained in the medium during TOP2 poison treatment.

#### Statistics.

Data are presented as the mean values ± S.E.M. values for *n* ≥ 3 replicate experiments; the number of replicates involved for each treatment is indicated within the figures. Statistical analysis was performed by one-way ANOVA with post hoc Tukey’s multiple comparison test. For signifying *P* values, * refers to *P* < 0.05, ** refers to *P* < 0.01, and *** refers to *P* < 0.001.

## Results

### 

#### MPO Inhibition Suppresses Etoposide-Induced TOP2A and TOP2B Covalent DNA Complex Formation in NB4 Cells.

**Fig. 1.**
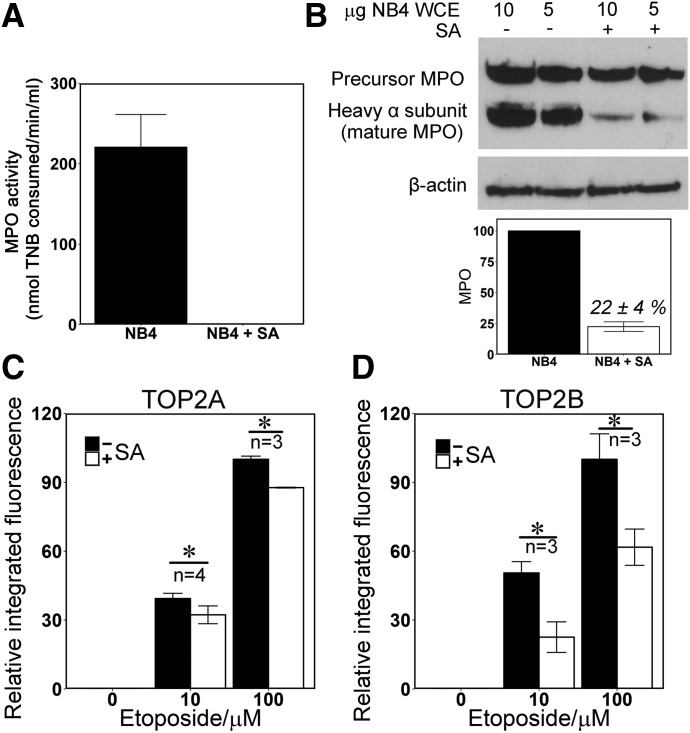
MPO inhibition reduces the level of TOP2-DNA covalent complexes formed by etoposide in NB4 cells. (A and B) Succinylacetone (SA) abolished MPO activity (A) and reduced mature protein levels in NB4 cells (B); NB4 cells were treated with 200 *µ*M SA for 48 hours and assayed for MPO activity (A) and MPO protein level by Western blotting of whole cell extracts (B). Blots were quantified by densitometry. (C and D) TOP2-DNA covalent complexes were quantified by TARDIS analysis using antibodies specific to TOP2A (C) or TOP2B (D). Integrated fluorescence values were determined per nucleus (at least 500 nuclei per treatment per replicate experiment). From these, median values were obtained for each treatment and means of the medians were calculated from replicate experiments (*n* = 3). Data are expressed as a percentage of the mean value obtained with 100 *μ*M etoposide in the absence of SA, ±S.E.M. Integrated fluorescence data corresponding to an individual experiment are also shown in Supplemental Fig. 3, A and B. *P < 0.05.

MPO activity can be efficiently reduced in cell culture systems by the heme biosynthesis inhibitor succinylacetone (SA) (Pinnix et al., 1994; Kagan et al., 2001; Vlasova et al., 2011). By using NB4 cells, an acute promyleocytic leukemia line that express MPO at a high level (Hu et al., 1993), we found that incubation with 200 *μ*M SA for 48 hours reduced MPO enzymatic activity to below the detection level of the assay employed, and as reported previously (Pinnix et al., 1994), it significantly depleted mature MPO protein (Fig. 1, A and B). Quantification of replicate blots indicated that mature MPO protein level was reduced to less than 25% of that in untreated cells. Notably, SA treatment of up to 72 hours did not affect TOP2A or TOP2B protein levels in NB4 cells, accelerate acidification of the medium, nor reduce cell viability or cell growth (Supplemental Fig. 2, B–E). Similarly, SA did not affect TOP2A or TOP2B enzymatic activity in vitro (Supplemental Fig. 2, F–H). We used the TARDIS assay (Willmore et al., 1998; Cowell et al., 2011b) to quantify stabilized TOP2-DNA covalent complexes in etoposide-treated cells. This assay employs sensitive quantitative immunofluorescence to analyze TOP2 content in agarose embedded “nuclear ghosts” that remain after noncovalently attached proteins and other cellular constituents have been removed from nuclear DNA by SDS-salt extraction (Supplemental Fig. 3, A and B). Pretreatment with SA significantly reduced the levels of etoposide-induced TOP2A and TOP2B-DNA complexes. This was true for both 10 and 100 *μ*M etoposide (Fig. 1, C and D; Supplemental Fig. 3, A and B). At both doses of etoposide, the magnitude of the affect was greater for TOP2B than for TOP2A. For TOP2B with 100 *μ*M etoposide SA pretreatment resulted in a 38% reduction in TOP2-complex formation, whereas the reduction was 12% for TOP2A. For 10 *μ*M etoposide, the respective figures were 55% and 18% (Fig. 1, C and D; Supplemental Table 1). As expected, SA pretreatment did not affect stabilized TOP2A or TOP2B-DNA complexes in K562 cells, a chronic myeloid leukemia-derived cell line that does not express MPO (Supplemental Fig. 3, C and D). Etoposide quinone displays enhanced TOP2-mediated DNA cleavage activity in vitro compared with the parent compound (Jacob et al., 2011), and this is more pronounced for TOP2B (Smith et al., 2014b). The observation that SA pretreatment, which reduces MPO-mediated etoposide phenoxy radical production, partially suppressed etoposide-induced TOP2-CC formation in MPO-expressing NB4 cells supports the conclusion that oxidative metabolism of etoposide plays a role in TOP2-mediated DNA damage in vivo.

#### MPO Expression in K562 Cells Stimulates Etoposide-Induced TOP2-DNA Covalent Complex Formation.

**Fig. 2.**
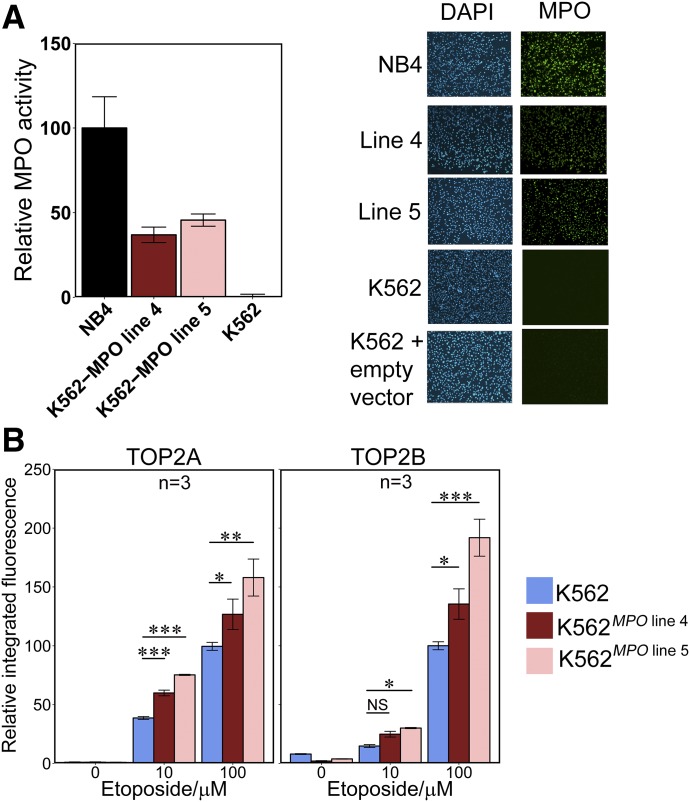
Expression of active MPO in K562 cells increases the level of etoposide-stabilized TOP2-DNA covalent complexes. (A) Comparison of MPO activity (left) and MPO immunofluorescence (right) in K562 cells, two MPO-expressing K562-derived cell lines (K562*^MPO^* ^line4^ K562*^MPO^* ^line5^), and in NB4 cells. (B) K562 and K562*^MPO^* cell lines were incubated with 10 or 100 *µ*M Etoposide or a vehicle control for 1 hour. Etoposide-stabilized TOP2-DNA complexes were quantified by TARDIS analysis using antibodies specific for TOP2A or TOP2B as described for Fig. 1. Numbers of replicates are indicated. *P < 0.05; **P < 0.01; ***P < 0.001.

K562 is a chronic myeloid leukemia derived cell line that does not express detectable MPO (Hu et al., 1993) (Supplemental Fig. 1). We transfected K562 cells with a human MPO expression construct and isolated clonal K562 lines expressing MPO. Two K562^MPO^ cell lines (MPO line 4 and MPO line 5) exhibited MPO activity at 37 ± 7.5% and 46 ± 6.0% of the level of NB4 cells, respectively. Both cell lines expressed MPO in all cells by immunofluorescence (Fig. 2A). Parental K562 cells and K562^MPO^ cell lines 4 and 5 were treated with 10 or 100 *µ*M etoposide and stabilized TOP2-DNA complexes were quantified. The K562^MPO^ cell lines displayed significantly higher levels of drug-stabilized complexes for both TOP2 isoforms compared with K562 cells lacking MPO expression at 100 *μ*M etoposide. At 10 *μ*M etoposide, raised levels of TOP2A and TOP2B stabilized complexes were observed with MPO line 5, and for line 4 significantly raised complex levels were observed only for TOP2A (Fig. 2B). After subtracting background complex levels, K562^MPO^ line 5 displayed a 1.6- and 2.0-fold increase in TOP2A- and TOP2B-DNA complexes, respectively, compared with K562 after 100 *μ*M etoposide treatment. For line 4, which expressed MPO at a lower level, a smaller fold increase in complexes was observed (Supplemental Table 1). By comparison, K562 cells transfected with an empty expression vector did not display increased TOP2 covalent complex formation (Supplemental Fig. 3, G and H). This further supports the notion that MPO-mediated activation contributes to etoposide-mediated DNA damage in MPO expressing cells.

#### MPO Contributes to Etoposide-Induced Cellular DNA Damage.

**Fig. 3.**
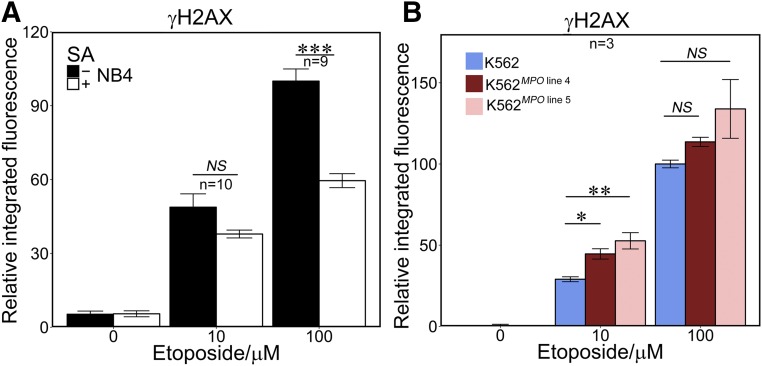
MPO activity results in raised levels of etoposide-induced H2AX phosphorylation. (A) NB4 cells were pretreated for 48 hours with SA (200 *µ*M) then incubated with etoposide (10 or 100 *µ*M). (B) K562 and K562*^MPO^* cell lines were incubated with 10 and 100 *µ*M etoposide or dimethyl sulfoxide vehicle control. For both (A) and (B), *γ*H2AX was quantified by immunofluorescence. Analysis was carried out as described for TARDIS analysis in Fig. 1. Data are expressed relative to the mean values obtained with 100 *μ*M etoposide in the absence of SA (A) or in wild-type parental K562 cells (B). Numbers of replicates are indicated. *P < 0.05; **P < 0.01; ***P < 0.001.

TOP2 poison-induced covalent DNA complexes are processed to DSBs that result in histone H2AX phosphorylation (Sunter et al., 2010; Cowell et al., 2011a, 2012) as part of a well-established response involving activation of the DNA damage kinase ataxia-telangiectasia mutated (Burma et al., 2001). Thus, *γ*H2AX can be used as a measure of DSBs generated by etoposide treatment. The inhibition of MPO using SA resulted in a 40% reduction in *γ*H2AX induced by 100 *µ*M etoposide (Fig. 3A; Supplemental Table 1). Notably, this is equivalent to the difference in *γ*H2AX signal observed between 10 and 100 *μ*M etoposide in the absence of SA. SA did not significantly affect the level of *γ*H2AX observed after exposure to 10 *μ*M etoposide. SA had no effect on etoposide-induced *γ*H2AX in K562 cells, which do not express MPO (Supplemental Fig. 3E).

Etoposide-induced *γ*H2AX formation was also examined in K562^MPO^ cell lines. For 10 *µ*M etoposide, MPO expression resulted in a significant rise in *γ*H2AX intensity (Fig. 3B), reaching a 1.8-fold increase over K562 cells for line 5 (Supplemental Table 1). At 100 *µ*M etoposide, neither cell line displayed a significant increase in *γ*H2AX over K562. However, the lack of significant effect of MPO expression when cells were treated with 100 *µ*M compared with the lower etoposide dose can be explained by the observation that *γ*H2AX formation reaches saturation at 100 *µ*M etoposide in K562 cells (Supplemental Fig. 3F).

K562 cells transfected with empty vector were also tested for induction of *γ*H2AX formation in comparison with nontransfected K562 cells. The empty vector control did not differ significantly from the nontransfected cells in *γ*H2AX signal formation upon etoposide treatment (Supplemental Fig. 3I). Thus, MPO activity results in elevated H2AX phosphorylation in etoposide-treated cells.

#### MPO Stimulates Mitoxantrone-Induced Accumulation of TOP2-DNA Complexes and H2AX Phosphorylation.

**Fig. 4.**
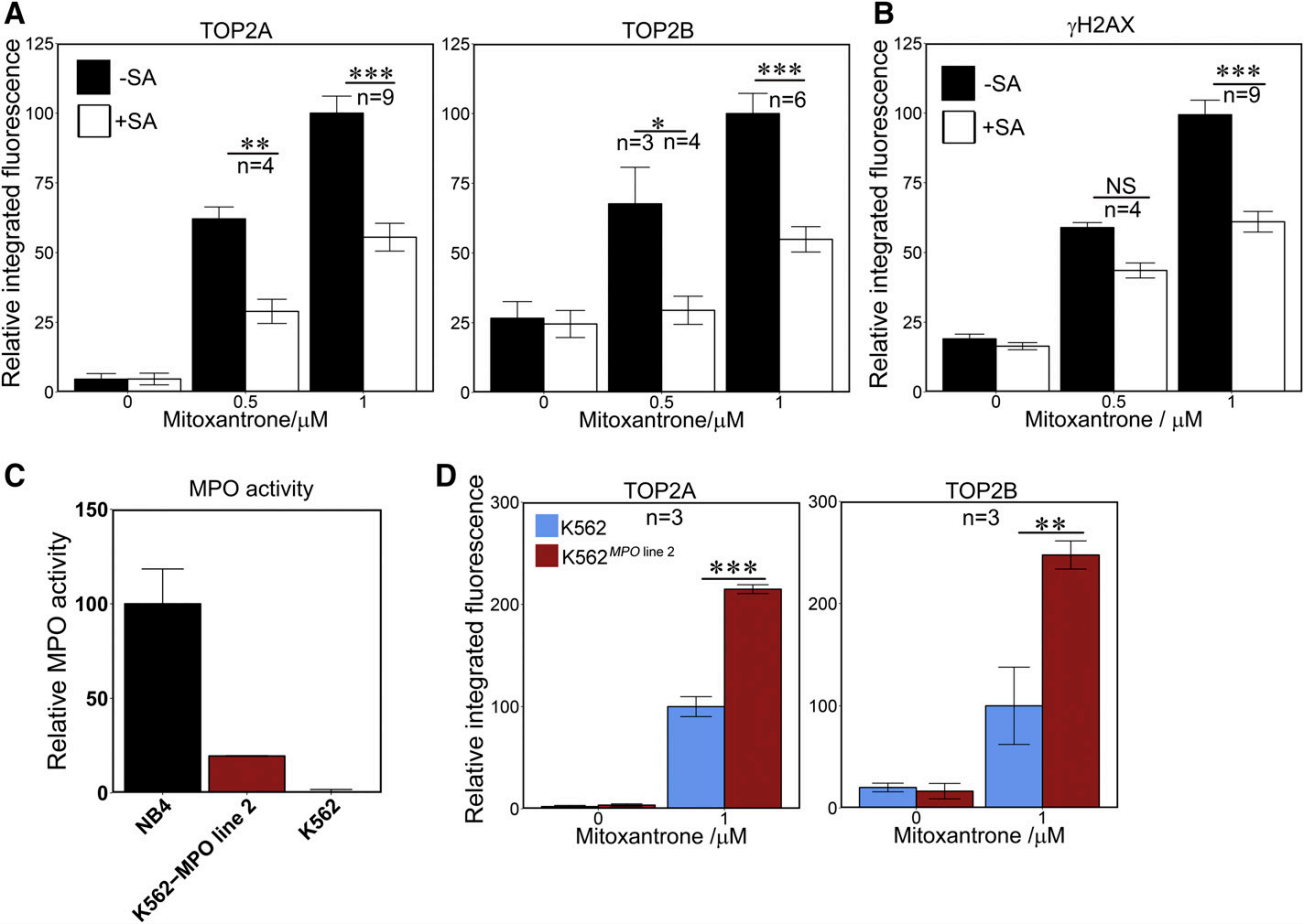
MPO activity enhances mitoxantrone-induced TOP2-DNA covalent complex formation and H2AX phosphorylation. (A and B) NB4 cells were pretreated with 200 *μ*M SA for 48 hours followed by a 1-hour incubation with 0.5 or 1 *μ*M mitoxantrone, or a vehicle control. TOP2-DNA covalent complexes and *γ*H2AX were quantified as in Figs. 1 and 3. Data are expressed relative to the mean values obtained with 1 *μ*M mitoxantrone in the absence of SA. (C) Quantification of MPO activity in K562*^MPO^* line 2 compared with NB4 and parental K562 cells. (D) MPO expression in K562 cells results in enhanced mitoxantrone-induced TOP2-DNA protein complex formation. Data are expressed relative to the mean value obtained for parental K562 cells treated with 1 *μ*M mitoxantrone. Numbers of replicates are indicated. *P < 0.05; **P < 0.01; ***P < 0.001.

The inhibitor SA, which effectively abolishes MPO activity in NB4 cells (Fig. 1), resulted in a substantial reduction in stabilized TOP2-DNA complexes induced by mitoxantrone (Fig. 4A). After subtraction of signal detected in control cells (0 *μ*M mitoxantrone), TOP2A and TOP2B complexes induced by 0.5 *μ*M mitoxantrone were reduced by 59 and 88%, respectively, whereas at 1 *μ*M mitoxantrone the figures were 43% and 63% (Supplemental Table 1). Similarly, SA pretreatment reduced the mitoxantrone induced *γ*H2AX signal by 39% for 1 *μ*M mitoxantrone (Fig. 4B).

K562^MPO^ cell line 2 expresses MPO activity at 20% of the level observed in NB4 cells (Fig. 4C). Mitoxantrone (1 *μ*M) induced 2.2- and 2.9-fold more stabilized TOP2A and TOP2B complexes in K562^MPO^ cell line 2 than in parental K562 cells (Fig. 4D; Supplemental Table 1).

Like etoposide, mitoxantrone is prone to oxidation by peroxidases such as MPO, and mitoxantrone can form DNA adducts via activation by formaldehyde (Panousis et al., 1995; Parker et al., 1999), but less is known about the impact of this on TOP2-DNA complex formation in cells or the downstream accumulation of DNA DSBs. The observed MPO activity requirement for maximal TOP2 complex formation as well as H2AX phosphorylation supports the idea that MPO-mediated oxidation directly or indirectly enhances the activity of mitoxantrone as a TOP2 poison.

#### Glutathione Depletion Stimulated Etoposide- and Mitoxantrone-Induced DNA Damage in an MPO-Dependent Manner.

**Fig. 5.**
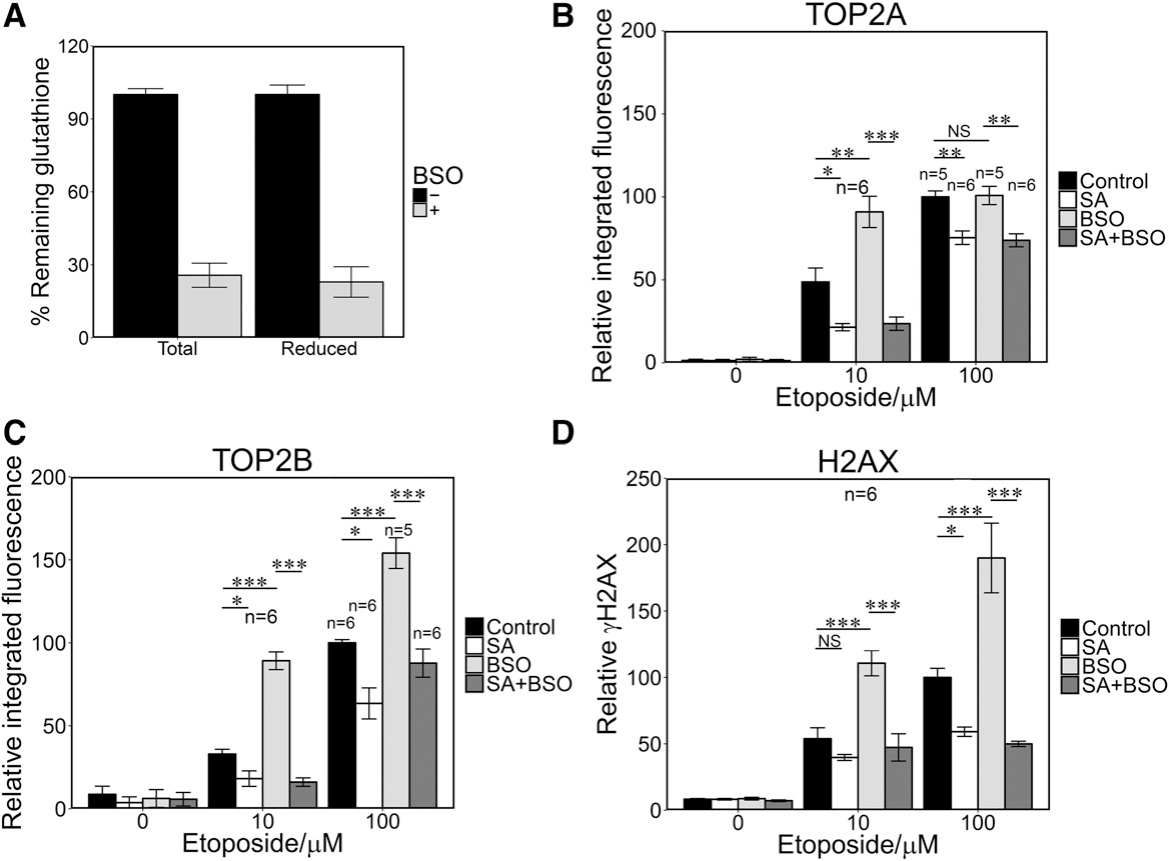
Glutathione depletion increases etoposide-mediated TOP2-DNA covalent complex formation and H2AX phosphorylation. (A) BSO preincubation (150 *μ*M, 4.5 hours) resulted in 70% reduction of total and reduced glutathione in NB4 cells. (B–D) NB4 cells were incubated in the presence or absence of SA (200 *μ*M) for 48 hours, BSO (150 *μ*M) for 4.5 hours, with both or with neither, followed by addition of 10 or 100 *μ*M etoposide for 1 hour. TARDIS and *γ*H2AX assays were performed as described in Figs 1 and 3. Numbers of replicates are indicated. *P < 0.05; **P < 0.01; ***P < 0.001.

**Fig. 6.**
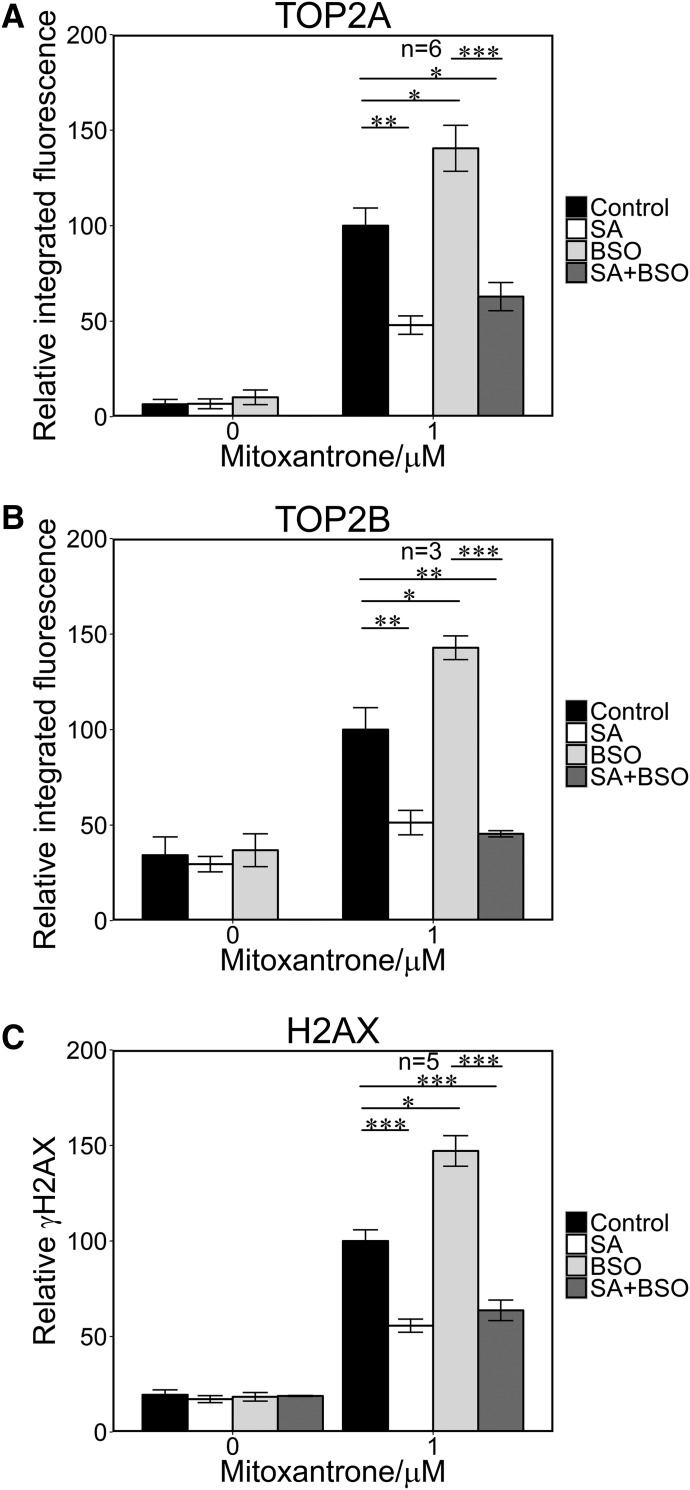
Glutathione depletion potentiates mitoxantrone-mediated TOP2 covalent complex formation and H2AX phosphorylation. NB4 cells were preincubated with SA, BSO, or both as described for Fig 4, followed by addition of 1 *μ*M mitoxantrone for 1 hour. TOP2A TARDIS (A), TOP2B TARDIS (B), and *γ*H2AX assays (C) were performed as described in Figs 1 and 3. *P < 0.05; **P < 0.01; ***P < 0.001.

Etoposide and mitoxantrone metabolites react with the low molecular weight thiol GSH (Yalowich et al., 1996; Panousis et al., 1997; Kagan et al., 2001; Fan et al., 2006). It follows that the effect of high MPO expression on TOP2 poison-mediated DNA damage in cells may therefore be limited by cellular GSH. To test this we used buthionine sulfoximine (BSO), which inhibits *γ*-glutamylcysteine synthetase and thus leads to GSH depletion in cells (Griffith, 1982). The addition of BSO did not affect TOP2 activity in in vitro activity assays (Supplemental Fig. 2, F–H). Glutathione levels were reduced by more than 70% after pretreatment with BSO (150 *μ*M, 4.5 hours; Fig. 5A). BSO-treated NB4 cells exhibited elevated levels of TOP2A- and TOP2B-DNA complexes induced by 10 *μ*M etoposide (1.9- and 3.4-fold, respectively), and for 100 *μ*M etoposide BSO increased TOP2B-DNA complexes (1.6 fold) but did not increase TOP2A-DNA complex levels (Fig. 5, B and C; Supplemental Table 2). In addition, BSO pretreatment significantly increased etoposide-induced H2AX phosphorylation at both doses of etoposide (2.2- and 2.0-fold increase, respectively; Fig. 5D; Supplemental Table 2). Significantly, BSO had no effect on TOP2 complexes or H2AX phosphorylation induced by etoposide when cells were pretreated with the MPO inhibitor SA. This is consistent with the previously described MPO-dependent (and therefore SA-suppressed) generation of etoposide quinone via the phenoxy-radical (Haim et al., 1986; Kagan et al., 2001), leading to elevated TOP2-DNA complex accumulation and histone H2AX phosphorylation due to elevated TOP2 poison activity (Gantchev and Hunting, 1998; Jacob et al., 2011). This activity is then enhanced under conditions of depleted glutathione, which would otherwise reduce the phenoxy radical and/or combine with etoposide quinone. BSO pretreatment also resulted in elevated mitoxantrone-induced stabilized TOP2-DNA complexes and H2AX phosphorylation (Fig. 6, A–C), although the magnitude of the effect was smaller than for etoposide (Fig. 5; Supplemental Table 3).

#### Direct Small Molecule Inhibitors of MPO Suppress Etoposide- and Mitoxantrone-Induced TOP2A and TOP2B Covalent DNA Complex Formation and H2AX Phosphorylation in NB4 Cells.

**Fig. 7.**
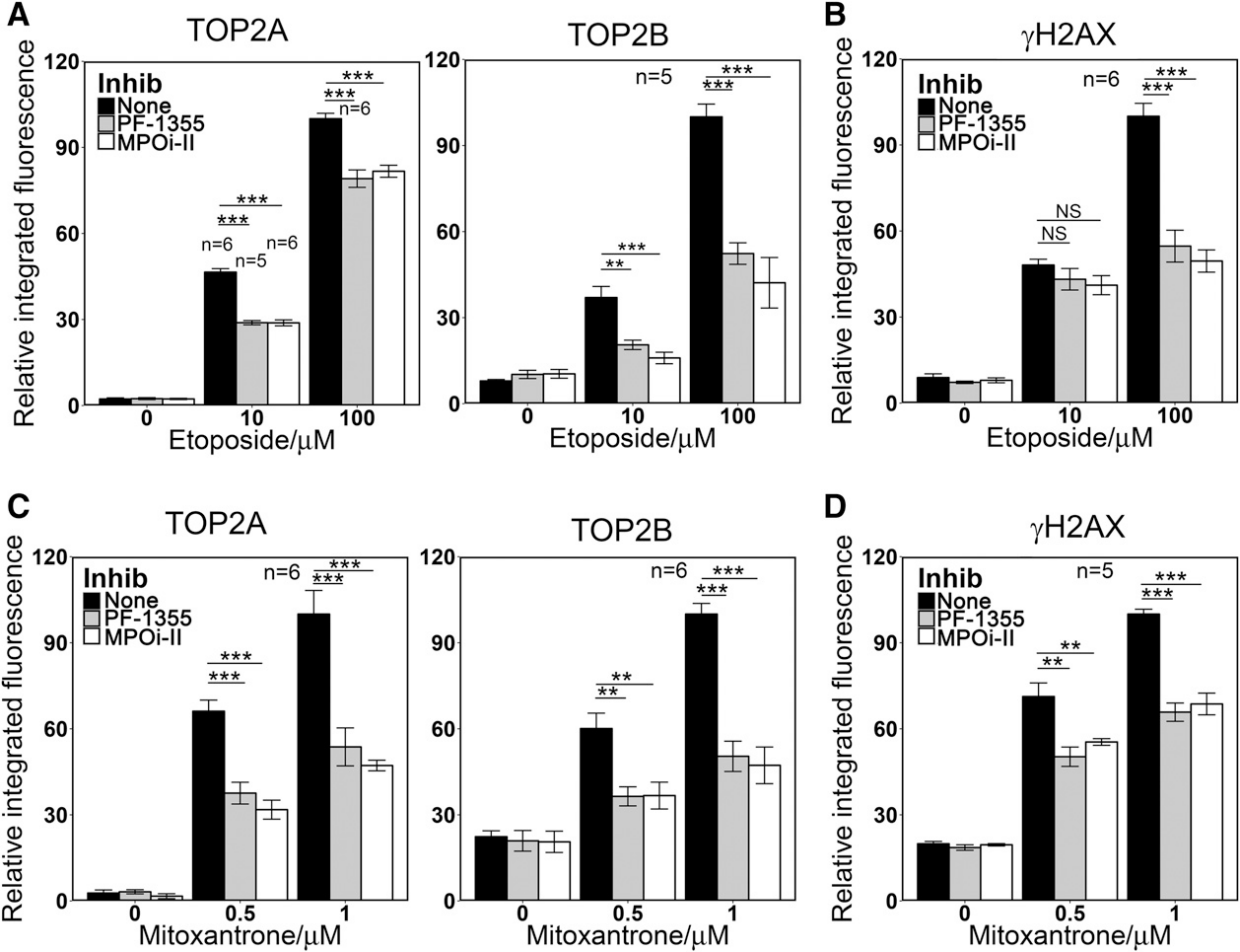
Direct MPO inhibition reduces the level of TOP2-DNA covalent complexes formation and H2AX phosphorylation induced by etoposide or mitoxantrone in NB4 cells. (A and B) NB4 cells were pretreated with MPO inhibitors PF-1355 (10 *μ*M, 4 hours) or MPOi-II (5 *μ*M, 4 hours) before adding 10 or 100 *μ*M etoposide or a vehicle control for 1 hour. (C and D**)** NB4 cells were pretreated with MPO inhibitors as in (A and B) before adding 0.5 or 1 *μ*M mitoxantrone or a vehicle control for 1 hour. TOP2-DNA covalent complexes (A and C) and *γ*H2AX (B and D) were quantified as in Figs 1 and 3. Numbers of replicates are indicated. **P < 0.01; ***P < 0.001.

The work described above employed the heme synthesis inhibitor SA that indirectly reduces cellular MPO activity. Recently a number of direct specific MPO inhibitors have been developed. These include PF-1355 (2-(6-(2,5-dimethoxyphenyl)-4-oxo-2-thioxo-3,4-dihydropyrimidin-1(2H)-yl)acetamide) (Zheng et al., 2015) and MPOi-II (4-(5-fluoro-1H-indol-3-yl)butanamide) (Soubhye et al., 2013). As expected, both of these inhibitors dramatically reduced MPO activity in NB4 cells (Supplemental Fig. 4). After 4 hours, MPO activity was undetectable in PF-1355-treated cells and for MPOi-II activity was reduced by more than 90%. Pretreatment with either inhibitor also reduced the levels of TOP2A and TOP2B DNA complexes and H2AX phosphorylation induced by etoposide or mitoxantrone in NB4 cells, having a greater effect on TOP2B (Fig. 7). The magnitudes of the effects were comparable to those observed with SA pretreatment (Supplemental Table 2; Figs. 1, C and D, 3A, and 4, A and B). As was observed for SA, PF-1355 and MPOi-II did not affect induction of TOP2A or TOP2B complexes or H2AX phosphorylation induced by etoposide or mitoxantrone in MPO nonexpressing K562 cells (Supplemental Fig. 4).

## Discussion

Therapy-related acute leukemia is a rare, but life threatening, complication of prior treatment of a primary cancer. Therefore, interventions that specifically protect myeloid cells from the unwanted effects of cytotoxic chemotherapies would be very welcome. MPO is expressed exclusively in cells of myeloid origin and is capable of enzymatic conversion of TOP2 poisons, including etoposide, mitoxantrone, and anthracyclines, to species with greater DNA damaging activity. So, in principle, MPO inhibition should partially protect myeloid precursors from TOP2 poison-mediated DNA damage, while preserving the desired cytotoxic effects in the target tumor cells. This idea is supported by the data reported here showing that three chemically distinct MPO inhibitors each reduce etoposide and mitoxantrone-induced TOP2-DNA complex and *γ*H2AX formation in NB4 cells, whereas conversely, exogenous MPO expression in K562 cells leads to increased etoposide- and mitoxantrone-induced damage.

Notably, the suppression of MPO activity had an approximately threefold greater effect on TOP2B complex formation than on TOP2A. The greater effect for TOP2B is of interest, because this TOP2 isoform appears to be required for the majority of etoposide-induced *MLL* and *RUNX1* chromosomal breaks (Cowell et al., 2012; Smith et al., 2014a) in a human lymphoblastoid cell line model and for etoposide-mediated carcinogenesis in a mouse model (Azarova et al., 2007). The MPO-dependent enhancement of the TOP2 poisoning activity of etoposide is likely to be due to production of etoposide phenoxy radicals and etoposide ortho-quinone in MPO-expressing cells (see Supplemental Fig. 2A). Etoposide phenoxy radicals have been observed in vitro and in cell culture systems (Haim et al., 1986; Kalyanaraman et al., 1989; Yalowich et al., 1996; Kagan et al., 2001; Vlasova et al., 2011) and can be further oxidized to etoposide quinone (Haim et al., 1986; Fan et al., 2006), which exhibits enhanced TOP2-dependent DNA cleavage activity compared with etoposide, especially for TOP2B (Jacob et al., 2011; Smith et al., 2014b). Etoposide ortho-quinone reacts spontaneously with GSH in solution (Fan et al., 2006), and so the effect of MPO expression on TOP2-DNA complex accumulation may be limited by GSH titration of etoposide quinone within cells. Indeed, we found that when GSH was depleted by BSO, treatment of NB4 cells with 10 *μ*M etoposide resulted in significantly elevated TOP2A- and TOP2B-DNA complex accumulation (1.9- and 3.4-fold, respectively), and this effect was abolished by SA pretreatment (Fig. 5). Notably, although glutathione depletion amplified the effect of SA on etoposide induced TOP2-DNA complexes, we were still able to measure suppression of etoposide-induced TOP2A and TOP2B complexes in the absence of BSO.

Although these data were mostly obtained with NB4 cells, which express MPO at a high level (Supplemental Fig. 1) (Hu et al., 1993), enhanced etoposide-induced TOP2-DNA complex formation and H2AX phosphorylation were also observed in K562 cells exogenously expressing MPO activity at less than 50% of the level of NB4 cells. For the analogous mitoxantrone experiments, exogenously expressed MPO resulted in a 1.6- and 2.9-fold increase in TOP2A- and TOP2B-DNA complexes, respectively, even though MPO was only expressed at 20% of the NB4 level. Thus, it appears that MPO can stimulate TOP2-mediated DNA damage even when expressed at moderate levels more similar to those that exist in bone marrow myeloid precursor cells.

While our results with etoposide in NB4 cells can be explained by etoposide redox activity in MPO expressing cells and the finding that etoposide quinone is a more effective TOP2 poison than its parent compound in vitro (Jacob et al., 2011; Smith et al., 2014b), the situation is less clear for mitoxantrone. Mitoxantrone can be oxidized by MPO, and products of this oxidation react covalently with DNA, both in cell-free systems and in cells (Panousis et al., 1995, 1997). Furthermore, mitoxantrone is activated by formaldehyde, also resulting in covalent interaction with DNA (Parker et al., 1999). However, it is currently unclear whether the metabolic products of mitoxantrone function as TOP2 poisons, and if so what their relative activity is compared with the parent compound. We show here that MPO downregulation significantly impairs mitoxantrone-induced TOP2-DNA complex accumulation in NB4 cells. Again the effect is larger for TOP2B than TOP2A. For 0.5 *μ*M mitoxantrone, SA preincubation decreased TOP2B-DNA complexes to a level that was no longer significantly above the background level obtained in the absence of mitoxantrone. Exogenous expression of MPO in K562 cells resulted in a more than doubling in TOP2A- and TOP2B-DNA complexes induced by mitoxantrone. Together, these data strongly suggest that oxidative metabolism of mitoxantrone leads to increased TOP2A and TOP2B poisoning. However, our data do not distinguish between direct enhanced poisoning of TOP2 by mitoxantrone metabolites, and generation of DNA adducts or repair intermediates that act as TOP2 poisons (Kingma et al., 1995; Sabourin and Osheroff, 2000).

As was observed for etoposide, GSH depletion increased the level of TOP2A- and TOP2B-DNA complexes induced by mitoxantrone consistent with the involvement of a metabolite that reacts readily with free thiols. Furthermore, high intensity chemotherapy leads to depletion of thiols including GSH (Jonas et al., 2000; Kasapović et al., 2010; Kadam and Abhang, 2013), and so the high level of TOP2 poison-induced DNA damage observed in BSO-treated cells is likely to reflect the generation of genetic lesions in bone marrow cells during cytotoxic chemotherapy regimens.

MPO has become a target of interest for novel anti-inflammatory drug development (Malle et al., 2007), and a number of potent specific MPO inhibitors were recently reported (Tidén et al., 2011; Forbes et al., 2013; Soubhye et al., 2013, 2016; Li et al., 2015; Zheng et al., 2015). We have used two such inhibitors, PF1355 and MPOi-II, and show that like SA, they reduce etoposide- and mitoxantrone-induced damage in MPO-expressing cells. Thus, novel compounds developed for a different clinical need could, in principle, be repurposed to reduce unwanted and carcinogenic TOP2 poison-induced genotoxic damage in critical bone marrow cells and could have a significant impact in the frequency of therapy-induced secondary leukemias.

## Abbreviations

AML: acute myeloid leukemia
BSO: buthionine sulfoximine
DAPI: 4’,6-diamidino-2-phenylindole
DSB: DNA double-strand break
GSH: reduced glutathione
H2AX: histone H2A.X
γH2AX: S-139 phospho histone H2A.X
MPO: myeloperoxidase
MPOi-II: 4-(5-fluoro-1H-indol-3-yl)butanamide
PF-1355: 2-(6-(2,5-dimethoxyphenyl)-4-oxo-2-thioxo-3,4-dihydropyrimidin-1(2H)-yl)acetamide
SA: succinylacetone
TARDIS: trapped in agarose DNA immunostaining assay
t-AML: therapy-related acute myeloid leukemia
TOP2: topoisomerase II
TOP2A: DNA topoisomerase II*β*
TOP2B: DNA topoisomerase II*β*
TOP2-CC: topoisomerase II covalent complex
